# Cardiovascular health in pediatric patients with X-linked hypophosphatemia under two years of burosumab therapy

**DOI:** 10.3389/fendo.2024.1400273

**Published:** 2024-05-16

**Authors:** Avivit Brener, Roxana Cleper, Guy Baruch, Ehud Rothschild, Michal Yackobovitch-Gavan, Gil Beer, Leonid Zeitlin, Livia Kapusta

**Affiliations:** ^1^ Institute of Pediatric Endocrinology and Diabetes, Dana-Dwek Children’s Hospital, Sourasky Medical Center, Tel Aviv, Israel; ^2^ Faculty of Medicine, Tel Aviv University, Tel Aviv, Israel; ^3^ The Pediatric Nephrology Unit, Dana-Dwek Children’s Hospital, Sourasky Medical Center, Tel Aviv, Israel; ^4^ Department of Internal Medicine, Sourasky Medical Center, Tel Aviv, Israel; ^5^ Department of Epidemiology and Preventive Medicine, School of Public Health, Faculty of Medicine, Tel Aviv University, Tel Aviv, Israel; ^6^ The Pediatric Cardiology Unit, Dana-Dwek Children’s Hospital, Sourasky Medical Center, Tel Aviv, Israel; ^7^ The Metabolic Bone Disease Unit, Pediatric Orthopedic Department, Dana-Dwek Children’s Hospital, Sourasky Medical Center, Tel Aviv, Israel; ^8^ Department of Pediatrics, Amalia Children’s Hospital, Radboud University Medical Centre, Nijmegen, Netherlands

**Keywords:** cardiac function, echocardiogram, elevated blood pressure, FGF23, left ventricular hypertrophy, X-linked hypophosphatemia

## Abstract

**Introduction:**

X-linked hypophosphatemia (XLH) is caused by an inactivating mutation in the phosphate-regulating endopeptidase X-linked (*PHEX*) gene whose defective product fails to control phosphatonin fibroblast growth factor 23 (FGF23) serum levels. Although elevated FGF23 levels have been linked with detrimental cardiac effects, the cardiologic outcomes in XLH patients have been subject to debate. Our study aimed to evaluate the prevalence and severity of cardiovascular morbidity in pediatric XLH patients before, during, and after a 2-year treatment period with burosumab, a recombinant anti-FGF23 antibody

**Methods:**

This prospective observational study was conducted in a tertiary medical center, and included 13 individuals with XLH (age range 0.6–16.2 years) who received burosumab every 2 weeks. Clinical assessment at treatment initiation and after .5, 1, and 2 years of uninterrupted treatment included anthropometric measurements and cardiologic evaluations (blood pressure [BP], electrocardiogram, conventional echocardiography, and myocardial strain imaging).

**Results:**

The linear growth of all patients improved significantly (mean height z-score: from -1.70 ± 0.80 to -0.96 ± 1.08, *P*=0.03). Other favorable effects were decline in overweight/obesity rates (from 46.2% to 23.1%) and decreased rates of elevated BP (systolic BP from 38.5% to 15.4%; diastolic BP from 38.5% to 23.1%). Electrocardiograms revealed no significant abnormality throughout the study period. Cardiac dimensions and myocardial strain parameters were within the normative range for age at baseline and remained unchanged during the study period.

**Conclusion:**

Cardiologic evaluations provided reassurance that 2 years of burosumab therapy did not cause cardiac morbidity. The beneficial effect of this treatment was a reduction in cardiovascular risk factors, as evidenced by the lower prevalence of both overweight/obesity and elevated BP.

## Introduction

An inactivating mutation in the phosphate-regulating endopeptidase homolog X-linked *(PHEX)* gene, either spontaneous or inherited, is the cause for X-linked hypophosphatemia (XLH; OMIM: 3307800) (1). A defective *PHEX* gene fails to curb the production of phosphatonin fibroblast growth factor 23 (FGF23) by osteoblasts and osteocytes (2). The upregulation of serum FGF23 results in hyperphosphaturia, hypophosphatemia, and decreased levels of calcitriol due to the inhibitory effect on renal 25-hydroxyvitamin D3 1-α-hydroxylase with the resultant hypo-mineralization of calcified tissues, such as bone and teeth (3). Children with XLH often experience impaired growth, rickets, bone deformations, and spontaneous dental abscesses in variable severity (4), and the main aim of treatment is alleviating these manifestations.

An often-disregarded aspect in childhood XLH, due to its clinical obscurity, is its impact on cardiometabolic health. Pediatric patients with XLH have increased prevalence of obesity (5) and evidence of unfavorable body composition (6), potentially placing them at risk for metabolic complications (7). Left ventricular hypertrophy (LVH) with several potential contributing factors has been reported in children affected by debilitating XLH (8). The chronic exposure to elevated levels of FGF23 have been proposed as a risk factor for cardiac morbidity, based upon its having been linked with cardiac hypertrophy in the context of chronic renal failure (9). In myocytes, FGF23 acts through FGF receptor 4 to promote pathological LVH, which ultimately increases the risk of heart function deterioration (10). In addition, the inhibition of 1,25-dihydroxyvitamin D by FGF23 has been suggested to promote the development of hypertension through the activation of the renin-angiotensin system (11). However, cardiovascular data on XLH adults with normal kidney function are inconsistent, and those data on children are scarce.

The main therapeutic strategy in pediatric XLH consists of alleviating the chronic hypophosphatemia. Until recently, patients received oral phosphate and vitamin D supplementation. In 2018, burosumab (Crysvita^®^, Ultragenyx), a recombinant anti-FGF23 monoclonal antibody, was introduced as a treatment for XLH (12), and in 2019 it was approved by the Israeli healthcare basket committee and has become the treatment of choice for pediatric patients with XLH. Data on the beneficial effects of this treatment on children’s growth and on their biochemical profile have been accumulating (13), but data on its impact on the cardiac architecture and function are lacking.

Standardized cardiac assessment consists of physical examination, blood pressure (BP) measurement, electrocardiogram (ECG), and conventional echocardiography. Myocardial strain imaging is another relatively new noninvasive echocardiographic modality for assessing global and regional myocardial deformation as a marker of subclinical cardiac disease (14, 15), and of early myocardial damage (even in the presence of relatively preserved fractional shortening or ejection fraction), for example, in monitoring chemotherapy-induced cardiotoxicity (16, 17). No previous studies on myocardial deformation in patients with XLH, with or without hypertension, have been conducted. In this prospective study, we aimed to explore the cardiovascular impact of burosumab treatment in pediatric XLH patients evaluated before, during, and after 2 years of treatment.

## Patients and methods

### Study design

This single-center prospective observational study was conducted in a multi-disciplined pediatric XLH clinic (endocrinology, metabolic bone disease, nephrology, and cardiology) in a tertiary medical center. The patients and/or their guardians gave informed consent to participate. The study protocol was approved by the Tel-Aviv Sourasky Medical Center Institutional Review Board (0778-20-TLV). The data were handled in accordance with the principles of GCP.

At study initiation, fifteen pediatric patients diagnosed with XLH have commenced burosumab treatment at the Pediatric Bone Clinic of Dana-Dwek Children’s Hospital, Tel-Aviv Sourasky Medical Center. The diagnosis of XLH was established by clinical, biochemical, and radiographic criteria, patients were also referred for genetic analysis for *PHEX* gene mutation confirmation. The patients were given explanations about potential cardiovascular complications related to XLH and were offered a comprehensive cardiologic evaluation, including electrocardiography, standardized echocardiography, and strain imaging. Burosumab was administered according to the recommended treatment protocol every 2 weeks, and the dose was adjusted (between 0.8-2 mg/kg) to achieve a serum phosphorus level at the lower normal range for age and healing of rickets (18). Oral phosphate supplement and calcitriol were discontinued 1 week before starting burosumab (19).

The study protocol consisted of multidiscipline clinic visits at selected time points during burosumab therapy (at treatment initiation and after .5, 1, and 2 years). Each visit included a physical examination, systolic and diastolic (BP) measurements, anthropometric measurements, Tanner pubertal stage determination, and laboratory evaluations. The cardiac evaluation (including a 12-lead ECG and detailed echocardiography) was performed at all meetings. The routine laboratory evaluation at each time point included serum concentrations of phosphate, calcium, creatinine, alkaline phosphatase, and intact parathyroid hormone.

### Clinical investigations

The blood pressure measurement using an automated monitor and the correct cuff size was conducted while the child was calmly seated. If abnormal blood pressure values were detected, the blood pressure measurement was repeated up to three times, with intervals between each measurement. BP percentiles were calculated with an online age-based pediatric BP calculator (20). Elevated systolic and diastolic BP were defined as BP percentiles above the 90^th^ percentile for sex, age, and height (21). Monitoring of growth involved measuring weight (wearing light clothing and by a standard calibrated scale) and height (by a commercial Harpenden-Holtain stadiometer). Body mass index (BMI) was then calculated as the weight in kilograms divided by the square of the height in meters. Anthropometric variables (height and BMI values) were converted to sex- and age-specific z-scores according to the CDC2000 Growth Charts for the United States (22). Overweight was defined as a 95^th^ >BMI percentile ≥85^th^ and obesity as BMI ≥95^th^, therefore overweight/obesity was categorized as BMI ≥85^th^ (BMI z-score ≥1.036) (23). The physical examination of the patients included pubertal stage assessment, which was performed according to Tanner and Marshall staging by a pediatric endocrinologist (24, 25). Patients were categorized as prepubertal (Tanner stage 1) or in-puberty (Tanner stages 2-5).

### Cardiologic evaluation

#### Electrocardiography

Resting 12-lead ECGs were scored for type of heart rhythm, heart rate, PR, QRS and QTc intervals, QRS axis and morphology of the P wave, QRS complex, and ST segment. Abnormal ECG findings were coded according to the Minnesota Code (26).

#### Echocardiograms

The echocardiograms were performed by an experienced technician and supervised by an experienced pediatric cardiologist (LK). Images were obtained with an 8.0 MHz or a 5.0 MHz phased-array transducer (depending upon the patient’s age and weight), using a locally available ultrasound machine (Siemens 2000, Germany). Quantification of cardiac chamber size, ventricular mass, and systolic and diastolic left ventricular function was measured in accordance with the recommendations for chamber quantification by the American Society of Echocardiography’s Guidelines and Standard Committee and the Chamber Quantification Writing Group (27). An M-mode ECG was performed in the short-axis views to measure the internal dimensions of the left ventricle at end-diastole (LVIDd) and end-systole (LVIDs), interventricular septum dimension in diastole (IVSd), and the posterior and septal wall thickness at end-diastole and end systole (LVPWd, IVSd, and LVPWs, respectively). Left ventricular mass (LVM) was calculated by means of the following formula: LVM = 0.8{1.04[({LVIDd + IVSd + LVPWd]3 − LVIDd3)]} + 0.6 (28) and then indexed (LVMI) by the body mass surface area (BSA). Z-scores for LVIDd, LVIDs, IVSd, and LVPWd were calculated using an online calculator (parameterz.com) based upon a healthy pediatric population (29). Left ventricular systolic function was determined using fractional shortening (FS), ejection fraction (EF), and rate-corrected velocity of circumferential fiber shortening (VCFc). FS was calculated by the following formula: (LVIDd-LVIDs)/LVIDd)/100. FS above 27% and ejection fraction (EF) 53% and higher were considered normal. VCFc was calculated with the formula of Colan et al. (30). The left ventricular diastolic function was evaluated using the early (E) and late (A) diastolic trans-mitral peak flow velocity ratio (E/A ratio), deceleration time of early filling velocity (Edec), and isovolumetric relaxation time (IVRT).

**Figure 1:**
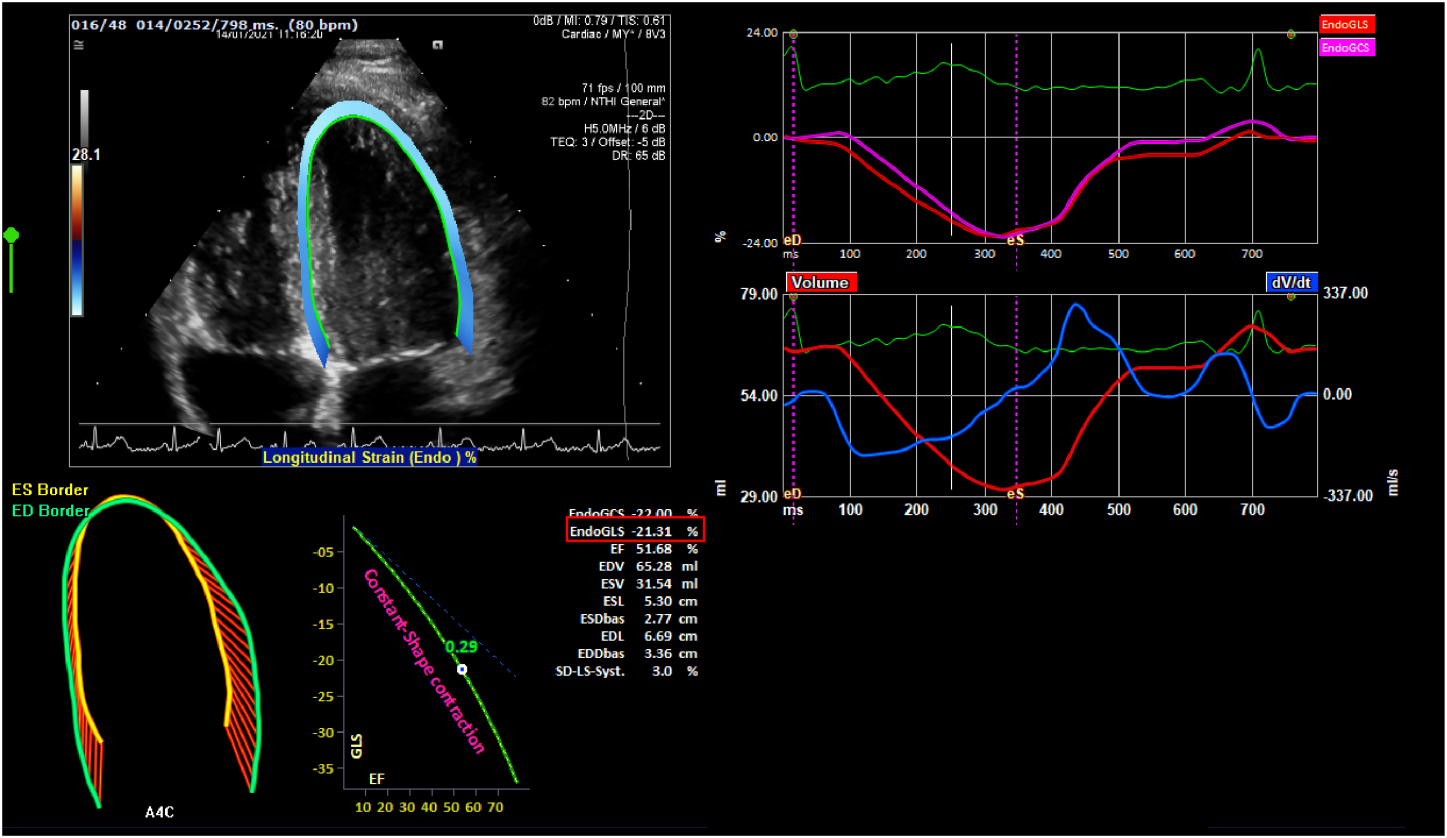
A depiction of strain echocardiography. The endocardial global longitudinal strain (endo GLS), represented as a percentage derived from the average of 6 segments of interest within the left ventricle, is highlighted in red.

Myocardial strain parameters were assessed by 2-dimensional (2D) Speckle tracking echocardiography image acquisition and off-line analysis (**Figure 1**). Three subsequent heartbeats of apical 4-chamber views of the LV and short axis images of the LV (at the papillary muscle level) were acquired at a frame rate of 60–90 frames/second. Images were analyzed using offline commercial feature-tracking software (2D CPA TomTec Imaging Systems, Germany) by 2 G.B. and E.R. who were blinded to the patient’s clinical and transthoracic ECG results. The myocardial strain analysis was performed according to methods previously described by our group (31). In short, one cardiac cycle with optimal image quality was selected. Endomyocardial borders of the LV were automatically traced throughout the cardiac cycle by the software with manual correction if necessary. For the LV, we evaluated peak and end-systolic global longitudinal strain (LVGLS, average of the basal septum, mid-septum, apical septum, apical lateral wall, mid-lateral wall, and basal lateral wall), peak and end-systolic GLS of the septal wall, peak and end-systolic GLS of the lateral wall, and peak systolic LV global circumferential strain (LVGCS: average of anterior septum, anterior-, lateral-, posterior-, inferior-, and septal walls at the papillary muscle level). Time-to-peak (TTP) for the systolic LVGLS and LVGCS (average of 6 segments, respectively) as well as for the septal and lateral walls (average of 3 segments, respectively) were calculated. All LV strain values are negative values, where a decrease in strain (indicting more positive value) is observed when LV function deteriorates.

### Statistical analysis

The data were analyzed with the Statistical Package for the Social Sciences software version 29 (SPSS Inc., Chicago, IL, USA). All statistical tests were 2-sided. The Kolmogorov-Smirnov test and the Shapiro-Wilk test were applied to test the normal distribution of continuous parameters. The data are expressed as means ± standard deviations (SDs) for normally distributed variables and median and intraquartile range (IQR) for abnormally distributed variables. The paired t-test was used for comparing the means of variables at baseline and after 2 years of burosumab therapy. Mixed linear regression models were used for analyzing the inter-individual’s change in each parameter from burosumab initiation, through .5, 1 and 2 years of treatment, 1 and 2 years of treatment. A *P* value of ≤.05 was considered significant.

## Results

The study included 13 patients with XLH who participated in all study meetings. At burosumab initiation, patients (10 girls and 3 boys) were 7.0 ± 5.6 years of age (range 0.6–16.2 years). They had been diagnosed with XLH at a mean age of 1.8 ± 1.0 years (range from birth to 3.5 years) and had been treated with conventional therapy of oral phosphate supplement and calcitriol for 5.2 ± 5.3 years before the initiation of burosumab.

The clinical and laboratory characteristics of the patients at burosumab initiation and after 2 years of treatment are presented in **Table 1**. The mean height z-score at baseline was -1.70 ± 0.80, with 7 (53.8%) patients scoring below the 3^rd^ percentile for height. The patients’ linear growth significantly improved under burosumab treatment, with a mean height z-score of -0.96 ± 1.08 after 2 years of treatment (*P* = .03), with 3 (23.1%) patients scoring below the 3^rd^ percentile. The mean BMI z-score did not change significantly throughout the 2 years of treatment. Six (46.2%) patients were classified with overweight/obesity at baseline. Following 2 years of burosumab therapy, only 3 (23.1%) of them were still classified as being overweight/obese. That reduction did not achieve a level of significance due to the small numbers of that group.

**Table 1:** Clinical and anthropometric parameters and laboratory evaluation at initiation and after 2 years of treatment with burosumab in patients with XLH.

	Burosumab Initiation	After 2 Years of Treatment	*P*-Value
Age, yr	7.0 ± 5.6	9.2 ± 5.7	
Anthropometric measurements			
Height (z-score)	-1.70 ± 0.80	-0.96 ± 1.08	**0.030**
BMI (z-score)	0.68 ± 0.82	0.27 ± 0.99	0.368
Blood pressure			
Systolic percentile	69.7 ± 25.5	72.6 ± 17.9	0.724
Elevated systolic, n (%)	5 (38.5)	2 (15.4)	0.378
Diastolic percentile	74.1 ± 25.9	64.6 ± 23.4	**0.004**
Elevated diastolic, n (%)	5 (38.5)	3 (23.1)	0.673
Pubertal stage			
Prepubertal, n (%)	10 (76.9)	8 (6.2)	0.673
In puberty, n (%)	3 (23.1)	5 (38.5)	
Laboratory evaluation			
Serum phosphate, mg/dL	2.82 ± 0.69	3.63 ± 0.64	**<0.001**
Serum calcium, mg/dL	9.66 ± 0.44	9.67 ± 0.44	0.994
Creatinine, (mg/dL)	0.36 ± 0.18	0.42 ± 0.14	0.317
Alkaline phosphatase, U/L	489.9 ± 175.2	279.4 ± 57.9	**<0.001**
PTH, pg/mL	47.9 ± 22.1	31.5 ± 22.8	**0.05**

BMI, body mass index, BP, blood pressure, PTH, parathyroid hormone. Normal range of PTH 6.7 – 38.8 pg/mL. The P value represents the paired t-test comparison between the value at burosumab initiation and after 2 years of treatment. Bold indicates significance.

The overall mean systolic and diastolic BP percentiles of patients were within normal limits at all study time points from baseline until 2 years of burosumab therapy. However, at the initiation of burosumab, 5 (38.5%) patients had elevated systolic BP and 5 (38.5%) patients had elevated diastolic BP (4 patients had elevated systolic and diastolic BP). The mean diastolic BP decreased significantly after 2 years of burosumab therapy (*P* = .004), while the systolic BP percentile remained unchanged (*P* = .724). The rates of elevated systolic and diastolic BP decreased after 2 years of burosumab therapy [n=2 (15.4%) and n=3 (23.1%), respectively].

None of the patients displayed abnormal cardiac auscultation at any of the assessment points throughout the entire study duration. The baseline ECGs of the XLH patients revealed 4 patients with an incomplete right bundle branch block (IRBBB). The prevalence of this minor abnormality decreased during follow-up and was identified in only 2 patients after 2 years of burosumab therapy. All 13 study patients at all time points showed sinus rhythm with normal conduction durations (PR interval, QRS interval, and QTc interval). There were no ST segment abnormalities. Based on ECG findings, none of the study patients met ECG criteria for left ventricular or right ventricular hypertrophy.

The ECG characteristics of the XLH patients at baseline and after 2 years of burosumab treatment are presented in **Table 2**. At baseline, mild cardiac anomalies were detected in 4 patients: they included mild and mild-to-moderated mitral regurgitation (MR) in 2 and a patent foramen ovale (PFO) in 2 (the latter at age 6 and 7 months). It is worth mentioning that although elevated BP was observed in 5 patients, no signs of LVH were identified in any participants at study initiation the cohort. While PFOs resolved in all the patients, MR persisted without deterioration. All z-scores of the LV dimensions were within normal at baseline and did not change significantly during the study period. The findings of mixed linear regression models assessing the inter-individual’s change in each parameter from burosumab initiation through 6 months, 1 and 2 years of treatment were insignificant for all of the LV dimensions.

**Table 2:** Conventional echocardiography and myocardial strain parameters in patients with XLH at initiation and after 2 years of treatment with burosumb.

Echocardiographic Parameter	Burosumab Initiation	After 2 Years of Treatment	*P*-value
Conventional echocardiography			
IVSd (z-score)	0.51 ± 1.24	0.02 ± 0.99	0.194
LVPWd (z-score)	-0.63 ± 0.99	-0.39 ± 1.12	0.524
LVPWs (z-score)	0.96 ± 0.26	1.09 ± 0.25	0.214
LVIDd (z-score)	1.09 ± 1.02	0.80 ± 1.02	0.507
LVIDs (z-score)	0.53 ± 1.11	0.32 ± 0.93	0.62
LVM (g)	54.2 ±37.1	75.6 ± 50.6	0.208
LVMI (g\*m* ^2^)	41.0 ± 10.9	38.7 ± 21.1	0.642
FS %	38.7 ± 4.1	38.4 ± 3.5	0.845
EF %	70.0 ± 5.3	69.1 ± 4.7	0.669
VCFc	43.4 ± 10.7	42.2 ± 7.9	0.781
IVRT (ms)	49.4 ± 12.7	52.7 ± 15.2	0.557
MV E/A ratio	1.76 ± 0.40	1.75 ± 0.28	0.908
Edec (ms)	93.5 ± 21.8	129.6 ± 35.4	0.206
Strain echocardiography			
Peak systolic LVGLS (%)	-23.6 ± 2.8	-23.2 ± 2.6	0.674
ES- LVGLS (%)	-23.2 ± 2.8	-23.0 ± 2.7	0.861
TTP systolic LVGLS (ms)	295.2 ± 32.5	297.2 ± 47.3	0.311
Peak systolic LVGCS (%)	-31.3 ± 2.2	-31.3 ± 1.9	0.49
ES- LVGCS (%)	-31.1 ± 2.2	-30.7 ± 1.9	0.408

IVSd= interventricular septum dimension in diastole ; LV, left ventricular; LVPWd and LVPWs= LV posterior wall dimension in diastole and systole, respectively; LVIDd and LVIDs, LV internal dimensions in diastole and systole, respectively; LVM, LV mass; LVMI, LV mass index; FS, fractional shortening; EF, ejection fraction; VCFc, rate-corrected velocity of circumferential fiber shortening; IVRT, isovolumetric relaxation time; Edec, deceleration time of early filling velocity; LVGLS, LV global longitudinal strain; ES, end systolic; TTP, time to peak; LVGCS, LV global circumferential strain; mm, millimeters; g, grams; m, meter; %, percentages; ms, milliseconds. P value represents the paired t-test comparison between the value at burosumab initiation and after 2 years of treatment. **Bold** indicates significance.

2D speckle tracking-derived myocardial strain parameters of patients with XLH are shown in **Table 2**. The longitudinal myocardial strain parameters and the GLS did not show any differences before and 2 years after burosumab therapy. Mixed linear regression models that assessed the inter-individual’s change in each parameter from the time of burosumab initiation, through 6 months, 1, and 2 years of treatment were insignificant for all of the LV strain parameters (data not shown).

## Discussion

In this prospective real-life study, we believe that we are the first to report on the findings in a comprehensive cardiac evaluation of pediatric patients with XLH, before, during, and after 2 years of burosumab therapy. It is reassuring that the thorough cardiologic assessment did not uncover any significant cardiac morbidity prior to the initiation of burosumab treatment, and that this status remained unchanged after 2 years of therapy. One beneficial effect of this treatment was a reduction in cardiovascular risk factors, as demonstrated by a reduction in the prevalence of both overweight and elevated BP.

When treating XLH with burosumab, the primary therapeutic mechanism involves reduction of the binding of excess FGF23 to its renal FGF receptors and the co-receptor klotho. This action alleviates the suppressive effects of the phosphaturic and 1,25-dihydroxyvitamin D induced by FGF23 (32). Burosumab could potentially yield further positive effects, given that persistently elevated FGF23 levels have the potential to negatively affect the cardiovascular system. A linear relationship had been described between FGF23 and cardiovascular mortality, independent of the chronic kidney disease status in a general adult population (33). This relationship was discovered to be age-dependent, thus underscoring the significance of klotho decline with aging in the damage caused by FGF23 (34). Furthermore, *in vivo* experiments in a klotho-deficient state demonstrated a direct cardiotoxic effect of elevated FGF23 levels (35).

The importance of the origin and dispersal pattern of FGF23 was notable as well. While an increase in circulating FGF23 did not directly affect the myocardium of mice with intact kidney function (36), the direct injection of recombinant FGF23 into their myocardium led to LVH, impaired vascular endothelial function, and the development of vascular calcifications (37). Clinical investigations examining cardiac morbidity in patients with XLH primarily concentrated on adults and yielded contradictory results (38, 39). No associations were observed between the levels of FGF23 in patients with XLH and cardiac morbidity (39). This suggests a distinct adverse impact of FGF23 in XLH, a condition characterized by hypophosphatemia, compared to its role in chronic kidney disease, a condition complicated by hyperphosphatemia, uremia, and volume overload.

Given that hypophosphatemia in XLH manifests from birth, it is crucial to ascertain the timing and contributing factors that lead to the onset of cardiac complications. Cross-sectional studies in pediatric patients have reported a higher incidence of hypertension, either with or without evidence of LVH (40, 41). The findings of our assessment before burosumab administration aligns with those of reports on the increased prevalence of elevated BP, although we did not detect any evidence of cardiac hypertrophy or dysfunction.

After 2 years of burosumab treatment, we observed a reduction in the diastolic BP percentiles and in the number of patients with elevated BP and that, reassuringly, cardiac structure and function remained unaffected. These favorable outcomes may be attributed to various factors. First, the administration of burosumab, an anti-FGF23 monoclonal antibody, arrested the continuous loss of phosphate, leading to the restoration of serum phosphate levels with a reduction in the compensatory increase in parathyroid hormone (PTH) levels. Supra-physiologic rise in PTH levels has been proposed as a factor contributing to progressive cardiac damage, with recovery observed upon resolution (42). Another favorable outcome was the improved weight status with a decreased overweight rate, which could potentially alleviate metabolic complications, including elevated BP. Finally, by counteracting FGF23, burosumab itself could contribute to the decrease observed in BP, in line with previous reports, suggesting an increase in klotho levels as a possible cardioprotective mechanism (43).

The present study has several limitations and strengths that bear mention. A major limitation is the relatively small study population, mainly due to the rare occurrence of the disease and inclusion limited to patients who were willing to undergo a cardiac evaluation at all time points. Another limitation is the absence of sex- and age-matched healthy control group to enable comparison. The major strengths of our study are its prospective design, the multi-disciplinary collaboration in the treatment and surveillance of our patients, and the uniformity of growth measurements, physical examinations, and cardiologic evaluations, including advanced imaging studies that were performed by trained medical personnel throughout the study.

In conclusion, the comprehensive cardiologic evaluation of pediatric patients with XLH did not reveal significant cardiac morbidity, although there was an increased prevalence of cardiovascular risk factors, such as elevated BP and overweight/obesity. There was a decrease in the prevalence of these risk factors over a 2-year period of treatment with burosumab, the anti-FGF23 antibody, while the functionality and structure of the heart remained intact. These findings demonstrate the favorable effects of this therapeutic approach in promoting the cardiovascular health of young patients with XLH.

## Data availability statement

The raw data supporting the conclusions of this article will be made available by the authors, without undue reservation.

## Ethics statement

The studies involving humans were approved by Tel Aviv Sourasky Medical Center. The studies were conducted in accordance with the local legislation and institutional requirements. Written informed consent for participation in this study was provided by the participants’ legal guardians/next of kin.

## Author contributions

AB: Writing – review & editing, Writing – original draft, Project administration, Methodology, Investigation, Formal analysis, Data curation, Conceptualization. RC: Writing – review & editing, Investigation, Data curation. GBa: Writing – review & editing, Software, Investigation, Data curation. ER: Writing – review & editing, Software, Investigation, Data curation. MY-G: Writing – review & editing, Formal analysis. GBe: Writing – review & editing, Software, Investigation, Data curation. LZ: Writing – review & editing, Methodology, Investigation, Data curation, Conceptualization. LK: Writing – review & editing, Supervision, Software, Methodology, Investigation, Data curation, Conceptualization.
